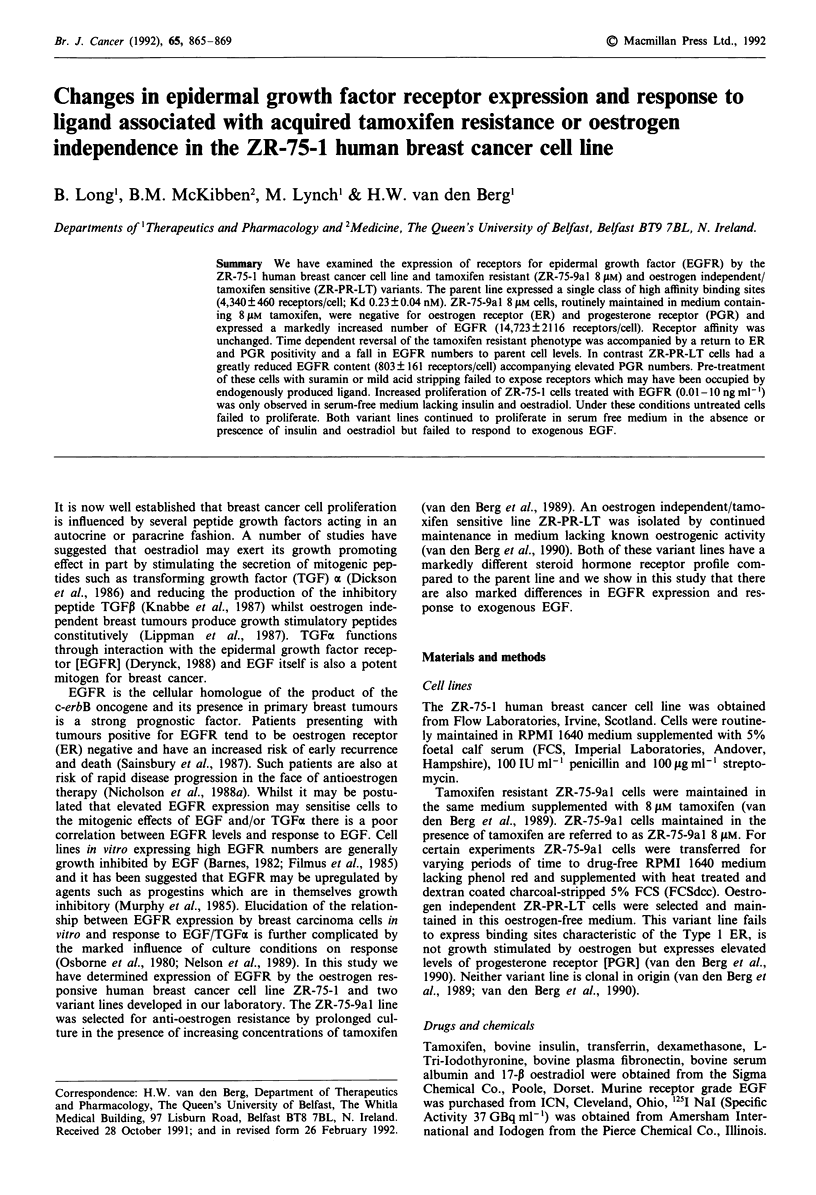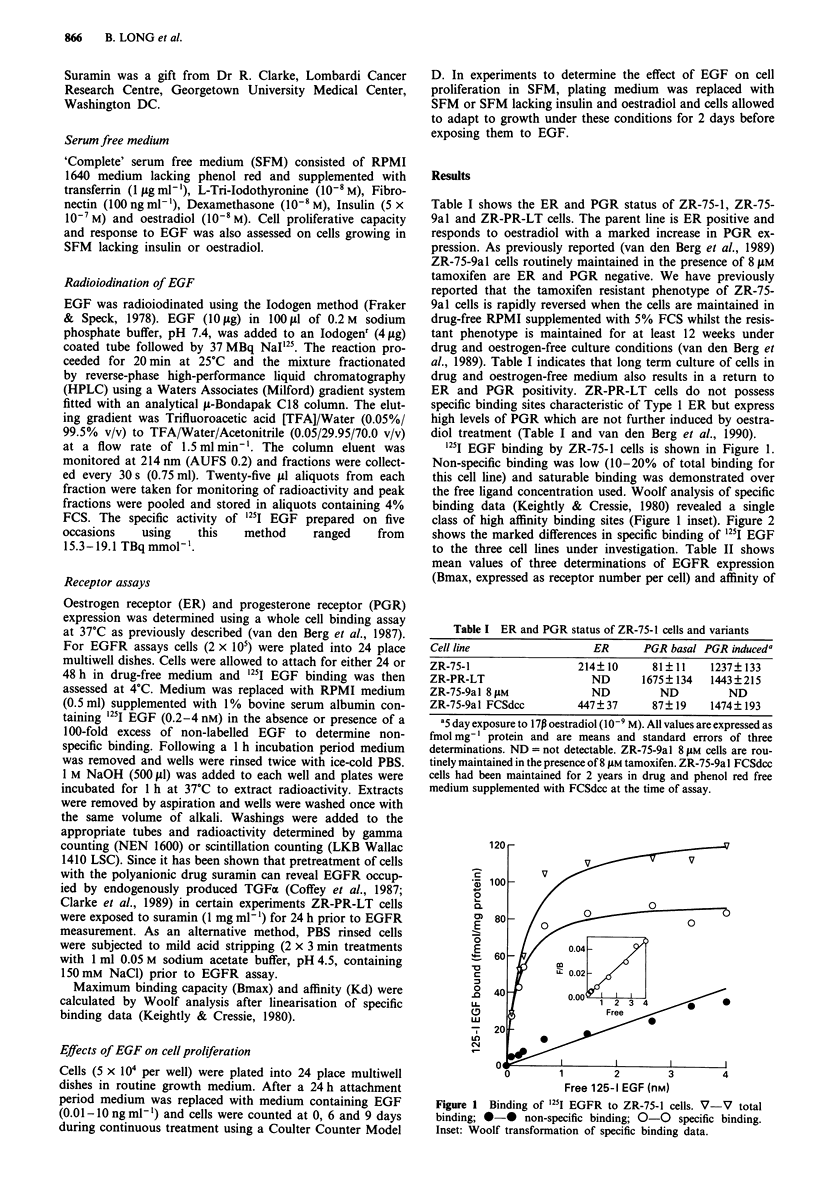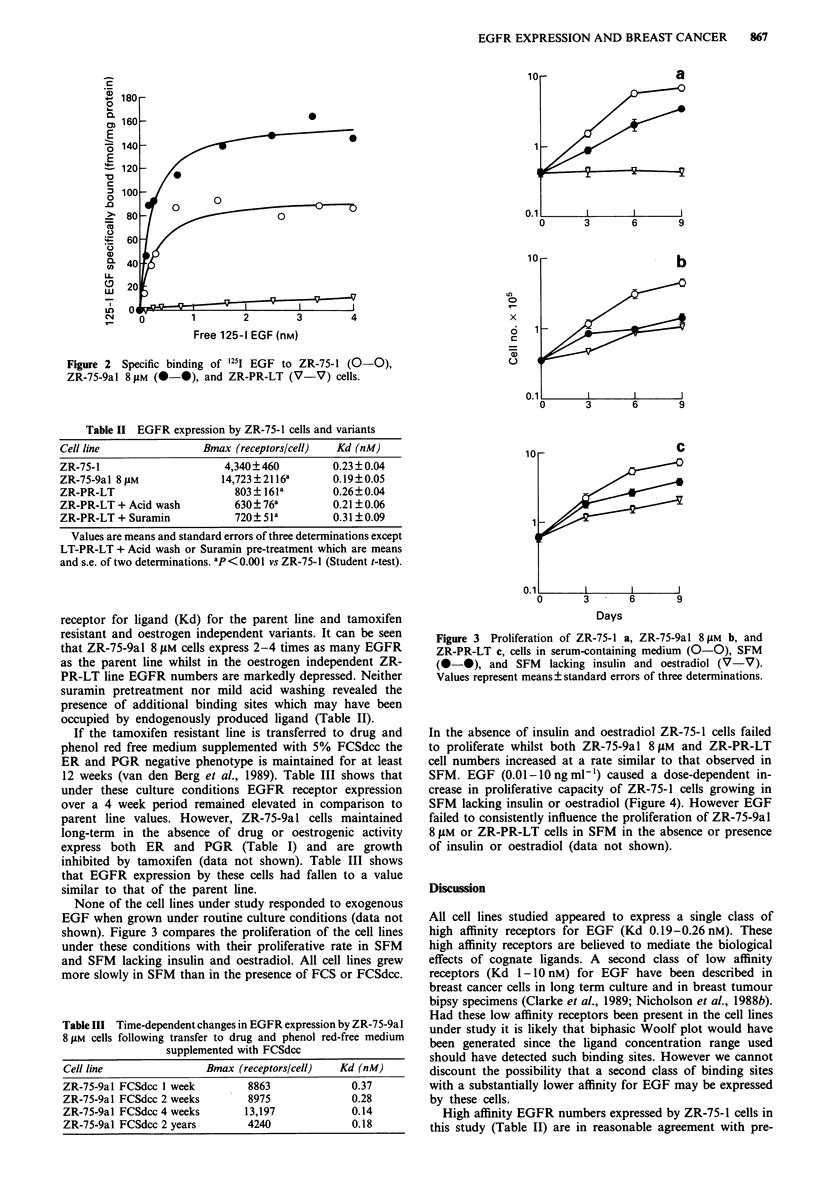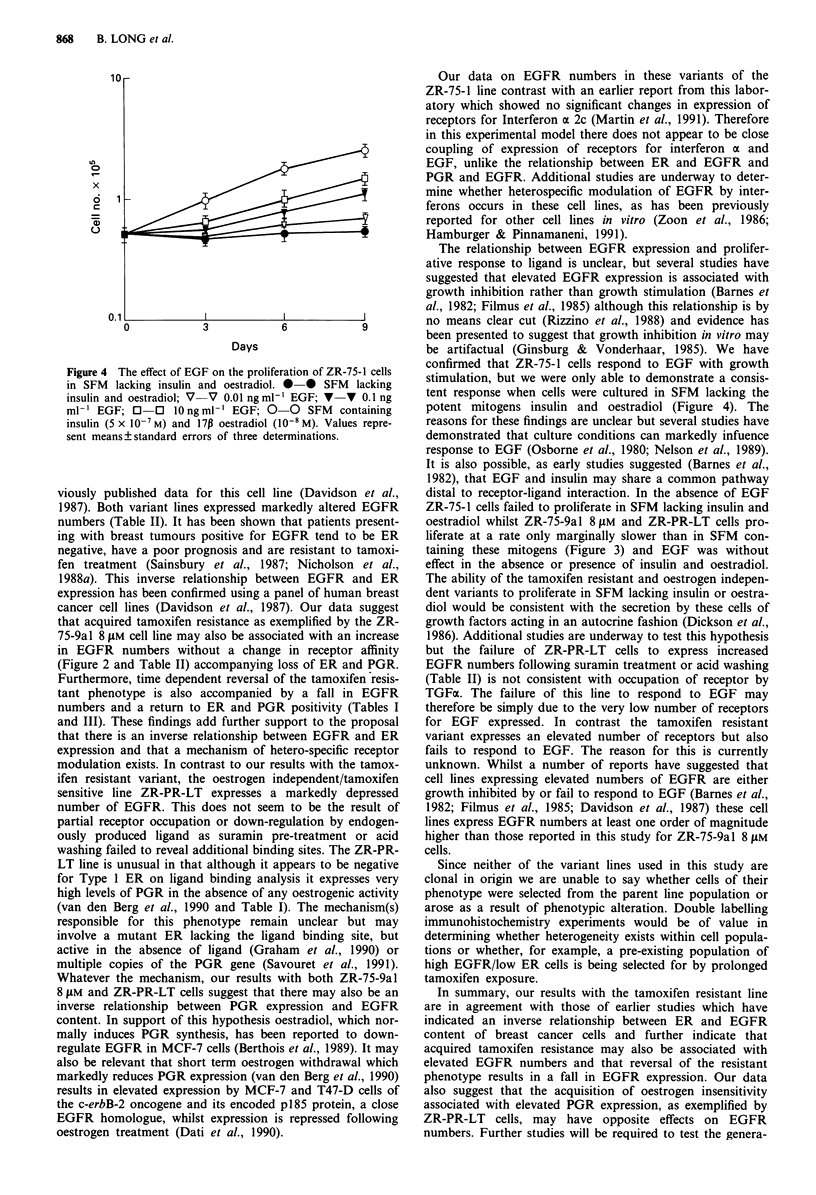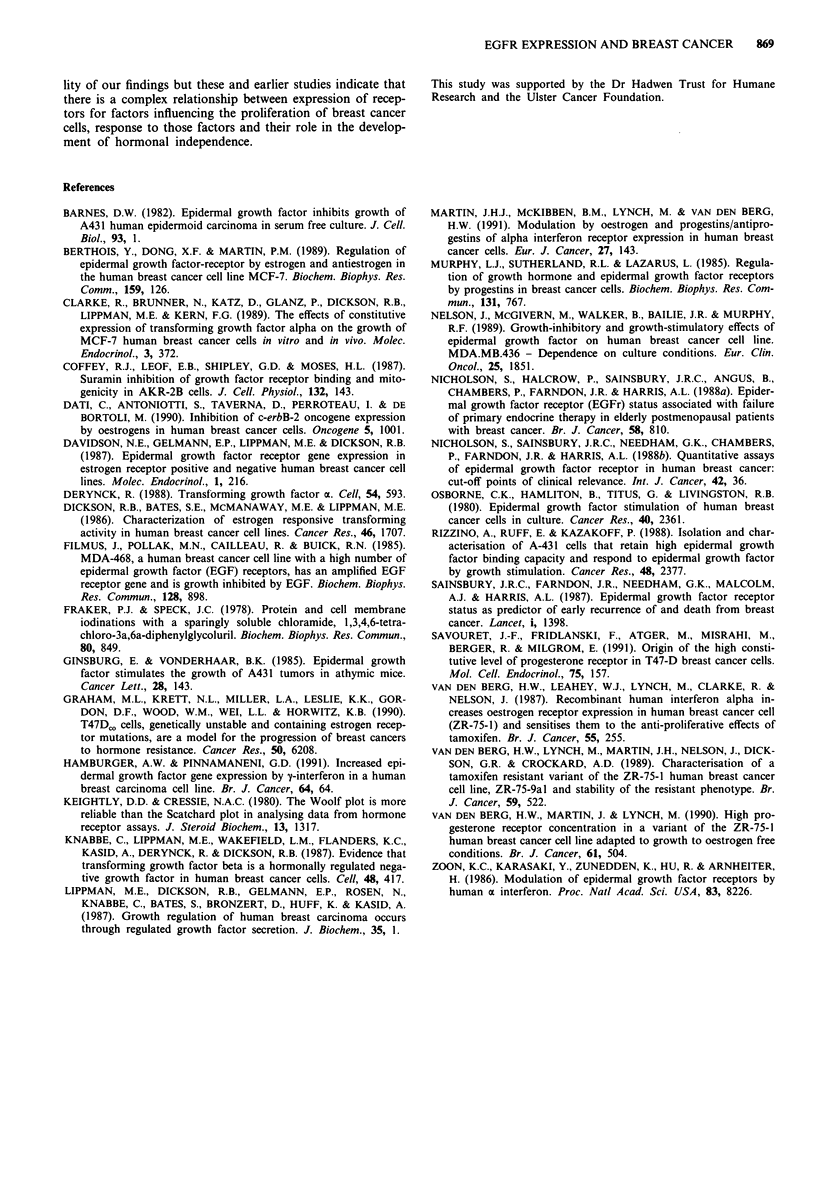# Changes in epidermal growth factor receptor expression and response to ligand associated with acquired tamoxifen resistance or oestrogen independence in the ZR-75-1 human breast cancer cell line.

**DOI:** 10.1038/bjc.1992.182

**Published:** 1992-06

**Authors:** B. Long, B. M. McKibben, M. Lynch, H. W. van den Berg

**Affiliations:** Department of Therapeutics and Pharmacology, Queen's University of Belfast, Ireland.

## Abstract

We have examined the expression of receptors for epidermal growth factor (EGFR) by the ZR-75-1 human breast cancer cell line and tamoxifen resistant (ZR-75-9al 8 microM) and oestrogen independent/tamoxifen sensitive (ZR-PR-LT) variants. The parent line expressed a single class of high affinity binding sites (4,340 +/- 460 receptors/cell; Kd 0.23 +/- 0.04 nM). ZR-75-9al 8 microM cells, routinely maintained in medium containing 8 microM tamoxifen, were negative for oestrogen receptor (ER) and progesterone receptor (PGR) and expressed a markedly increased number of EGFR (14,723 +/- 2116 receptors/cell). Receptor affinity was unchanged. Time dependent reversal of the tamoxifen resistant phenotype was accompanied by a return to ER and PGR positivity and a fall in EGFR numbers to parent cell levels. In contrast ZR-PR-LT cells had a greatly reduced EGFR content (803 +/- 161 receptors/cell) accompanying elevated PGR numbers. Pre-treatment of these cells with suramin or mild acid stripping failed to expose receptors which may have been occupied by endogenously produced ligand. Increased proliferation of ZR-75-1 cells treated with EGFR (0.01-10 ng ml-1) was only observed in serum-free medium lacking insulin and oestradiol. Under these conditions untreated cells failed to proliferate. Both variant lines continued to proliferate in serum free medium in the absence or presence of insulin and oestradiol but failed to respond to exogenous EGF.


Br. J. Cancer (1992), 65, 865-869 © Macmillan Press Ltd., 1992

### Changes in epidermal growth factor receptor expression and response to ligand associated with acquired tamoxifen resistance or oestrogen independence in the ZR-75-1 human breast cancer cell line

B. Long[1], B.M. McKibben[2], M. Lynch[1] & H.W. van den Berg[1]

*Departments of [1]Therapeutics and Pharmacology and [2]Medicine, The Queen's University of Belfast, Belfast BT9 7BL, N. Ireland.*

**Summary** We have examined the expression of receptors for epidermal growth factor (EGFR) by the ZR-75-1 human breast cancer cell line and tamoxifen resistant (ZR-75-9a1 8 μM) and oestrogen independent/tamoxifen sensitive (ZR-PR-LT) variants. The parent line expressed a single class of high affinity binding sites (4,340 ± 460 receptors/cell; Kd 0.23 ± 0.04 nM). ZR-75-9a1 8 μM cells, routinely maintained in medium containing 8 μM tamoxifen, were negative for oestrogen receptor (ER) and progesterone receptor (PGR) and expressed a markedly increased number of EGFR (14,723 ± 2116 receptors/cell). Receptor affinity was unchanged. Time dependent reversal of the tamoxifen resistant phenotype was accompanied by a return to ER and PGR positivity and a fall in EGFR numbers to parent cell levels. In contrast ZR-PR-LT cells had a greatly reduced EGFR content (803 ± 161 receptors/cell) accompanying elevated PGR numbers. Pre-treatment of these cells with suramin or mild acid stripping failed to expose receptors which may have been occupied by endogenously produced ligand. Increased proliferation of ZR-75-1 cells treated with EGFR (0.01 – 10 ng $ml^{-1}$) was only observed in serum-free medium lacking insulin and oestradiol. Under these conditions untreated cells failed to proliferate. Both variant lines continued to proliferate in serum free medium in the absence or prescence of insulin and oestradiol but failed to respond to exogenous EGF.

It is now well established that breast cancer cell proliferation is influenced by several peptide growth factors acting in an autocrine or paracrine fashion. A number of studies have suggested that oestradiol may exert its growth promoting effect in part by stimulating the secretion of mitogenic peptides such as transforming growth factor (TGF) α (Dickson *et al.*, 1986) and reducing the production of the inhibitory peptide TGFβ (Knabbe *et al.*, 1987) whilst oestrogen independent breast tumours produce growth stimulatory peptides constitutively (Lippman *et al.*, 1987). TGFα functions through interaction with the epidermal growth factor receptor [EGFR] (Derynck, 1988) and EGF itself is also a potent mitogen for breast cancer.

EGFR is the cellular homologue of the product of the c-*erb*B oncogene and its presence in primary breast tumours is a strong prognostic factor. Patients presenting with tumours positive for EGFR tend to be oestrogen receptor (ER) negative and have an increased risk of early recurrence and death (Sainsbury *et al.*, 1987). Such patients are also at risk of rapid disease progression in the face of antioestrogen therapy (Nicholson *et al.*, 1988*a*). Whilst it may be postulated that elevated EGFR expression may sensitise cells to the mitogenic effects of EGF and/or TGFα there is a poor correlation between EGFR levels and response to EGF. Cell lines *in vitro* expressing high EGFR numbers are generally growth inhibited by EGF (Barnes, 1982; Filmus *et al.*, 1985) and it has been suggested that EGFR may be upregulated by agents such as progestins which are in themselves growth inhibitory (Murphy *et al.*, 1985). Elucidation of the relationship between EGFR expression by breast carcinoma cells *in vitro* and response to EGF/TGFα is further complicated by the marked influence of culture conditions on response (Osborne *et al.*, 1980; Nelson *et al.*, 1989). In this study we have determined expression of EGFR by the oestrogen responsive human breast cancer cell line ZR-75-1 and two variant lines developed in our laboratory. The ZR-75-9a1 line was selected for anti-oestrogen resistance by prolonged culture in the presence of increasing concentrations of tamoxifen (van den Berg *et al.*, 1989). An oestrogen independent/tamoxifen sensitive line ZR-PR-LT was isolated by continued maintenance in medium lacking known oestrogenic activity (van den Berg *et al.*, 1990). Both of these variant lines have a markedly different steroid hormone receptor profile compared to the parent line and we show in this study that there are also marked differences in EGFR expression and response to exogenous EGF.

Correspondence: H.W. van den Berg, Department of Therapeutics and Pharmacology, The Queen's University of Belfast, The Whitla Medical Building, 97 Lisburn Road, Belfast BT8 7BL, N. Ireland.
Received 28 October 1991; and in revised form 26 February 1992.

### Materials and methods

### *Cell lines*

The ZR-75-1 human breast cancer cell line was obtained from Flow Laboratories, Irvine, Scotland. Cells were routinely maintained in RPMI 1640 medium supplemented with 5% foetal calf serum (FCS, Imperial Laboratories, Andover, Hampshire), 100 IU $ml^{-1}$ penicillin and 100 μg $ml^{-1}$ streptomycin.

Tamoxifen resistant ZR-75-9a1 cells were maintained in the same medium supplemented with 8 μM tamoxifen (van den Berg *et al.*, 1989). ZR-75-9a1 cells maintained in the presence of tamoxifen are referred to as ZR-75-9a1 8 μM. For certain experiments ZR-75-9a1 cells were transferred for varying periods of time to drug-free RPMI 1640 medium lacking phenol red and supplemented with heat treated and dextran coated charcoal-stripped 5% FCS (FCSdcc). Oestrogen independent ZR-PR-LT cells were selected and maintained in this oestrogen-free medium. This variant line fails to express binding sites characteristic of the Type 1 ER, is not growth stimulated by oestrogen but expresses elevated levels of progesterone receptor [PGR] (van den Berg *et al.*, 1990). Neither variant line is clonal in origin (van den Berg *et al.*, 1989; van den Berg *et al.*, 1990).

### *Drugs and chemicals*

Tamoxifen, bovine insulin, transferrin, dexamethasone, L-Tri-Iodothyronine, bovine plasma fibronectin, bovine serum albumin and 17-β oestradiol were obtained from the Sigma Chemical Co., Poole, Dorset. Murine receptor grade EGF was purchased from ICN, Cleveland, Ohio, $^{125}$I NaI (Specific Activity 37 GBq $ml^{-1}$) was obtained from Amersham International and Iodogen from the Pierce Chemical Co., Illinois.

Suramin was a gift from Dr R. Clarke, Lombardi Cancer Research Centre, Georgetown University Medical Center, Washington DC.

*Serum free medium*

'Complete' serum free medium (SFM) consisted of RPMI 1640 medium lacking phenol red and supplemented with transferrin (1 $\mu g\,ml^{-1}$), L-Tri-Iodothyronine ($10^{-8}$ M), Fibronectin (100 $ng\,ml^{-1}$), Dexamethasone ($10^{-8}$ M), Insulin ($5 \times 10^{-7}$ M) and oestradiol ($10^{-8}$ M). Cell proliferative capacity and response to EGF was also assessed on cells growing in SFM lacking insulin or oestradiol.

*Radioiodination of EGF*

EGF was radioiodinated using the Iodogen method (Fraker & Speck, 1978). EGF (10 $\mu$g) in 100 $\mu$l of 0.2 M sodium phosphate buffer, pH 7.4, was added to an Iodogen[r] (4 $\mu$g) coated tube followed by 37 MBq $NaI^{125}$. The reaction proceeded for 20 min at 25°C and the mixture fractionated by reverse-phase high-performance liquid chromatography (HPLC) using a Waters Associates (Milford) gradient system fitted with an analytical $\mu$-Bondapak C18 column. The eluting gradient was Trifluoroacetic acid [TFA]/Water (0.05%/99.5% v/v) to TFA/Water/Acetonitrile (0.05/29.95/70.0 v/v) at a flow rate of 1.5 $ml\,min^{-1}$. The column eluent was monitored at 214 nm (AUFS 0.2) and fractions were collected every 30 s (0.75 ml). Twenty-five $\mu$l aliquots from each fraction were taken for monitoring of radioactivity and peak fractions were pooled and stored in aliquots containing 4% FCS. The specific activity of $^{125}$I EGF prepared on five occasions using this method ranged from 15.3–19.1 TBq $mmol^{-1}$.

*Receptor assays*

Oestrogen receptor (ER) and progesterone receptor (PGR) expression was determined using a whole cell binding assay at 37°C as previously described (van den Berg et al., 1987). For EGFR assays cells ($2 \times 10^5$) were plated into 24 place multiwell dishes. Cells were allowed to attach for either 24 or 48 h in drug-free medium and $^{125}$I EGF binding was then assessed at 4°C. Medium was replaced with RPMI medium (0.5 ml) supplemented with 1% bovine serum albumin containing $^{125}$I EGF (0.2–4 nM) in the absence or presence of a 100-fold excess of non-labelled EGF to determine non-specific binding. Following a 1 h incubation period medium was removed and wells were rinsed twice with ice-cold PBS. 1 M NaOH (500 $\mu$l) was added to each well and plates were incubated for 1 h at 37°C to extract radioactivity. Extracts were removed by aspiration and wells were washed once with the same volume of alkali. Washings were added to the appropriate tubes and radioactivity determined by gamma counting (NEN 1600) or scintillation counting (LKB Wallac 1410 LSC). Since it has been shown that pretreatment of cells with the polyanionic drug suramin can reveal EGFR occupied by endogenously produced TGFα (Coffey et al., 1987; Clarke et al., 1989) in certain experiments ZR-PR-LT cells were exposed to suramin (1 $mg\,ml^{-1}$) for 24 h prior to EGFR measurement. As an alternative method, PBS rinsed cells were subjected to mild acid stripping ($2 \times 3$ min treatments with 1 ml 0.05 M sodium acetate buffer, pH 4.5, containing 150 mM NaCl) prior to EGFR assay.

Maximum binding capacity (Bmax) and affinity (Kd) were calculated by Woolf analysis after linearisation of specific binding data (Keightly & Cressie, 1980).

*Effects of EGF on cell proliferation*

Cells ($5 \times 10^4$ per well) were plated into 24 place multiwell dishes in routine growth medium. After a 24 h attachment period medium was replaced with medium containing EGF (0.01–10 $ng\,ml^{-1}$) and cells were counted at 0, 6 and 9 days during continuous treatment using a Coulter Counter Model D. In experiments to determine the effect of EGF on cell proliferation in SFM, plating medium was replaced with SFM or SFM lacking insulin and oestradiol and cells allowed to adapt to growth under these conditions for 2 days before exposing them to EGF.

### Results

Table I shows the ER and PGR status of ZR-75-1, ZR-75-9a1 and ZR-PR-LT cells. The parent line is ER positive and responds to oestradiol with a marked increase in PGR expression. As previously reported (van den Berg et al., 1989) ZR-75-9a1 cells routinely maintained in the presence of 8 $\mu$M tamoxifen are ER and PGR negative. We have previously reported that the tamoxifen resistant phenotype of ZR-75-9a1 cells is rapidly reversed when the cells are maintained in drug-free RPMI supplemented with 5% FCS whilst the resistant phenotype is maintained for at least 12 weeks under drug and oestrogen-free culture conditions (van den Berg et al., 1989). Table I indicates that long term culture of cells in drug and oestrogen-free medium also results in a return to ER and PGR positivity. ZR-PR-LT cells do not possess specific binding sites characteristic of Type 1 ER but express high levels of PGR which are not further induced by oestradiol treatment (Table I and van den Berg et al., 1990).

$^{125}$I EGF binding by ZR-75-1 cells is shown in Figure 1. Non-specific binding was low (10–20% of total binding for this cell line) and saturable binding was demonstrated over the free ligand concentration used. Woolf analysis of specific binding data (Keightly & Cressie, 1980) revealed a single class of high affinity binding sites (Figure 1 inset). Figure 2 shows the marked differences in specific binding of $^{125}$I EGF to the three cell lines under investigation. Table II shows mean values of three determinations of EGFR expression (Bmax, expressed as receptor number per cell) and affinity of

**Table I** ER and PGR status of ZR-75-1 cells and variants

| *Cell line* | *ER* | *PGR basal* | *PGR induced*[a] |
|---|---|---|---|
| ZR-75-1 | 214±10 | 81±11 | 1237±133 |
| ZR-PR-LT | ND | 1675±134 | 1443±215 |
| ZR-75-9a1 8 $\mu$M | ND | ND | ND |
| ZR-75-9a1 FCSdcc | 447±37 | 87±19 | 1474±193 |

[a]5 day exposure to 17β oestradiol ($10^{-9}$ M). All values are expressed as fmol $mg^{-1}$ protein and are means and standard errors of three determinations. ND = not detectable. ZR-75-9a1 8 $\mu$M cells are routinely maintained in the presence of 8 $\mu$M tamoxifen. ZR-75-9a1 FCSdcc cells had been maintained for 2 years in drug and phenol red free medium supplemented with FCSdcc at the time of assay.

**Figure 1** Binding of $^{125}$I EGFR to ZR-75-1 cells. ∇—∇ total binding; ●—● non-specific binding; O—O specific binding. Inset: Woolf transformation of specific binding data.

**Figure 2** Specific binding of $^{125}$I EGF to ZR-75-1 (O—O), ZR-75-9a1 8 μM (●—●), and ZR-PR-LT (∇—∇) cells.

**Table II** EGFR expression by ZR-75-1 cells and variants

| *Cell line* | *Bmax (receptors/cell)* | *Kd (nM)* |
|---|---|---|
| ZR-75-1 | 4,340±460 | 0.23±0.04 |
| ZR-75-9a1 8 μM | 14,723±2116[a] | 0.19±0.05 |
| ZR-PR-LT | 803±161[a] | 0.26±0.04 |
| ZR-PR-LT + Acid wash | 630±76[a] | 0.21±0.06 |
| ZR-PR-LT + Suramin | 720±51[a] | 0.31±0.09 |

Values are means and standard errors of three determinations except LT-PR-LT + Acid wash or Suramin pre-treatment which are means and s.e. of two determinations. [a]$P<0.001$ *vs* ZR-75-1 (Student *t*-test).

receptor for ligand (Kd) for the parent line and tamoxifen resistant and oestrogen independent variants. It can be seen that ZR-75-9a1 8 μM cells express 2–4 times as many EGFR as the parent line whilst in the oestrogen independent ZR-PR-LT line EGFR numbers are markedly depressed. Neither suramin pretreatment nor mild acid washing revealed the presence of additional binding sites which may have been occupied by endogenously produced ligand (Table II).

If the tamoxifen resistant line is transferred to drug and phenol red free medium supplemented with 5% FCSdcc the ER and PGR negative phenotype is maintained for at least 12 weeks (van den Berg *et al.*, 1989). Table III shows that under these culture conditions EGFR receptor expression over a 4 week period remained elevated in comparison to parent line values. However, ZR-75-9a1 cells maintained long-term in the absence of drug or oestrogenic activity express both ER and PGR (Table I) and are growth inhibited by tamoxifen (data not shown). Table III shows that EGFR expression by these cells had fallen to a value similar to that of the parent line.

None of the cell lines under study responded to exogenous EGF when grown under routine culture conditions (data not shown). Figure 3 compares the proliferation of the cell lines under these conditions with their proliferative rate in SFM and SFM lacking insulin and oestradiol. All cell lines grew more slowly in SFM than in the presence of FCS or FCSdcc.

**Table III** Time-dependent changes in EGFR expression by ZR-75-9a1 8 μM cells following transfer to drug and phenol red-free medium supplemented with FCSdcc

| *Cell line* | *Bmax (receptors/cell)* | *Kd (nM)* |
|---|---|---|
| ZR-75-9a1 FCSdcc 1 week | 8863 | 0.37 |
| ZR-75-9a1 FCSdcc 2 weeks | 8975 | 0.28 |
| ZR-75-9a1 FCSdcc 4 weeks | 13,197 | 0.14 |
| ZR-75-9a1 FCSdcc 2 years | 4240 | 0.18 |

**Figure 3** Proliferation of ZR-75-1 **a**, ZR-75-9a1 8 μM **b**, and ZR-PR-LT **c**, cells in serum-containing medium (O—O), SFM (●—●), and SFM lacking insulin and oestradiol (∇—∇). Values represent means±standard errors of three determinations.

In the absence of insulin and oestradiol ZR-75-1 cells failed to proliferate whilst both ZR-75-9a1 8 μM and ZR-PR-LT cell numbers increased at a rate similar to that observed in SFM. EGF (0.01–10 ng ml$^{-1}$) caused a dose-dependent increase in proliferative capacity of ZR-75-1 cells growing in SFM lacking insulin or oestradiol (Figure 4). However EGF failed to consistently influence the proliferation of ZR-75-9a1 8 μM or ZR-PR-LT cells in SFM in the absence or presence of insulin or oestradiol (data not shown).

### Discussion

All cell lines studied appeared to express a single class of high affinity receptors for EGF (Kd 0.19–0.26 nM). These high affinity receptors are believed to mediate the biological effects of cognate ligands. A second class of low affinity receptors (Kd 1–10 nM) for EGF have been described in breast cancer cells in long term culture and in breast tumour bipsy specimens (Clarke *et al.*, 1989; Nicholson *et al.*, 1988*b*). Had these low affinity receptors been present in the cell lines under study it is likely that biphasic Woolf plot would have been generated since the ligand concentration range used should have detected such binding sites. However we cannot discount the possibility that a second class of binding sites with a substantially lower affinity for EGF may be expressed by these cells.

High affinity EGFR numbers expressed by ZR-75-1 cells in this study (Table II) are in reasonable agreement with pre-

**Figure 4** The effect of EGF on the proliferation of ZR-75-1 cells in SFM lacking insulin and oestradiol. ●—● SFM lacking insulin and oestradiol; ▽—▽ 0.01 ng $ml^{-1}$ EGF; ▼—▼ 0.1 ng $ml^{-1}$ EGF; □—□ 10 ng $ml^{-1}$ EGF; ○—○ SFM containing insulin ($5 \times 10^{-7}$ M) and 17β oestradiol ($10^{-8}$ M). Values represent means ± standard errors of three determinations.

viously published data for this cell line (Davidson et al., 1987). Both variant lines expressed markedly altered EGFR numbers (Table II). It has been shown that patients presenting with breast tumours positive for EGFR tend to be ER negative, have a poor prognosis and are resistant to tamoxifen treatment (Sainsbury et al., 1987; Nicholson et al., 1988a). This inverse relationship between EGFR and ER expression has been confirmed using a panel of human breast cancer cell lines (Davidson et al., 1987). Our data suggest that acquired tamoxifen resistance as exemplified by the ZR-75-9a1 8 μM cell line may also be associated with an increase in EGFR numbers without a change in receptor affinity (Figure 2 and Table II) accompanying loss of ER and PGR. Furthermore, time dependent reversal of the tamoxifen resistant phenotype is also accompanied by a fall in EGFR numbers and a return to ER and PGR positivity (Tables I and III). These findings add further support to the proposal that there is an inverse relationship between EGFR and ER expression and that a mechanism of hetero-specific receptor modulation exists. In contrast to our results with the tamoxifen resistant variant, the oestrogen independent/tamoxifen sensitive line ZR-PR-LT expresses a markedly depressed number of EGFR. This does not seem to be the result of partial receptor occupation or down-regulation by endogenously produced ligand as suramin pre-treatment or acid washing failed to reveal additional binding sites. The ZR-PR-LT line is unusual in that although it appears to be negative for Type 1 ER on ligand binding analysis it expresses very high levels of PGR in the absence of any oestrogenic activity (van den Berg et al., 1990 and Table I). The mechanism(s) responsible for this phenotype remain unclear but may involve a mutant ER lacking the ligand binding site, but active in the absence of ligand (Graham et al., 1990) or multiple copies of the PGR gene (Savouret et al., 1991). Whatever the mechanism, our results with both ZR-75-9a1 8 μM and ZR-PR-LT cells suggest that there may also be an inverse relationship between PGR expression and EGFR content. In support of this hypothesis oestradiol, which normally induces PGR synthesis, has been reported to down-regulate EGFR in MCF-7 cells (Berthois et al., 1989). It may also be relevant that short term oestrogen withdrawal which markedly reduces PGR expression (van den Berg et al., 1990) results in elevated expression by MCF-7 and T47-D cells of the c-erbB-2 oncogene and its encoded p185 protein, a close EGFR homologue, whilst expression is repressed following oestrogen treatment (Dati et al., 1990).

Our data on EGFR numbers in these variants of the ZR-75-1 line contrast with an earlier report from this laboratory which showed no significant changes in expression of receptors for Interferon α 2c (Martin et al., 1991). Therefore in this experimental model there does not appear to be close coupling of expression of receptors for interferon α and EGF, unlike the relationship between ER and EGFR and PGR and EGFR. Additional studies are underway to determine whether heterospecific modulation of EGFR by interferons occurs in these cell lines, as has been previously reported for other cell lines in vitro (Zoon et al., 1986; Hamburger & Pinnamaneni, 1991).

The relationship between EGFR expression and proliferative response to ligand is unclear, but several studies have suggested that elevated EGFR expression is associated with growth inhibition rather than growth stimulation (Barnes et al., 1982; Filmus et al., 1985) although this relationship is by no means clear cut (Rizzino et al., 1988) and evidence has been presented to suggest that growth inhibition in vitro may be artifactual (Ginsburg & Vonderhaar, 1985). We have confirmed that ZR-75-1 cells respond to EGF with growth stimulation, but we were only able to demonstrate a consistent response when cells were cultured in SFM lacking the potent mitogens insulin and oestradiol (Figure 4). The reasons for these findings are unclear but several studies have demonstrated that culture conditions can markedly infuence response to EGF (Osborne et al., 1980; Nelson et al., 1989). It is also possible, as early studies suggested (Barnes et al., 1982), that EGF and insulin may share a common pathway distal to receptor-ligand interaction. In the absence of EGF ZR-75-1 cells failed to proliferate in SFM lacking insulin and oestradiol whilst ZR-75-9a1 8 μM and ZR-PR-LT cells proliferate at a rate only marginally slower than in SFM containing these mitogens (Figure 3) and EGF was without effect in the absence or presence of insulin and oestradiol. The ability of the tamoxifen resistant and oestrogen independent variants to proliferate in SFM lacking insulin or oestradiol would be consistent with the secretion by these cells of growth factors acting in an autocrine fashion (Dickson et al., 1986). Additional studies are underway to test this hypothesis but the failure of ZR-PR-LT cells to express increased EGFR numbers following suramin treatment or acid washing (Table II) is not consistent with occupation of receptor by TGFα. The failure of this line to respond to EGF may therefore be simply due to the very low number of receptors for EGF expressed. In contrast the tamoxifen resistant variant expresses an elevated number of receptors but also fails to respond to EGF. The reason for this is currently unknown. Whilst a number of reports have suggested that cell lines expressing elevated numbers of EGFR are either growth inhibited by or fail to respond to EGF (Barnes et al., 1982; Filmus et al., 1985; Davidson et al., 1987) these cell lines express EGFR numbers at least one order of magnitude higher than those reported in this study for ZR-75-9a1 8 μM cells.

Since neither of the variant lines used in this study are clonal in origin we are unable to say whether cells of their phenotype were selected from the parent line population or arose as a result of phenotypic alteration. Double labelling immunohistochemistry experiments would be of value in determining whether heterogeneity exists within cell populations or whether, for example, a pre-existing population of high EGFR/low ER cells is being selected for by prolonged tamoxifen exposure.

In summary, our results with the tamoxifen resistant line are in agreement with those of earlier studies which have indicated an inverse relationship between ER and EGFR content of breast cancer cells and further indicate that acquired tamoxifen resistance may also be associated with elevated EGFR numbers and that reversal of the resistant phenotype results in a fall in EGFR expression. Our data also suggest that the acquisition of oestrogen insensitivity associated with elevated PGR expression, as exemplified by ZR-PR-LT cells, may have opposite effects on EGFR numbers. Further studies will be required to test the generality of our findings but these and earlier studies indicate that there is a complex relationship between expression of receptors for factors influencing the proliferation of breast cancer cells, response to those factors and their role in the development of hormonal independence.

This study was supported by the Dr Hadwen Trust for Humane Research and the Ulster Cancer Foundation.

### References

BARNES, D.W. (1982). Epidermal growth factor inhibits growth of A431 human epidermoid carcinoma in serum free culture. *J. Cell. Biol.*, **93,** 1.

BERTHOIS, Y., DONG, X.F. & MARTIN, P.M. (1989). Regulation of epidermal growth factor-receptor by estrogen and antiestrogen in the human breast cancer cell line MCF-7. *Biochem. Biophys. Res. Comm.*, **159,** 126.

CLARKE, R., BRUNNER, N., KATZ, D., GLANZ, P., DICKSON, R.B., LIPPMAN, M.E. & KERN, F.G. (1989). The effects of constitutive expression of transforming growth factor alpha on the growth of MCF-7 human breast cancer cells *in vitro* and *in vivo*. *Molec. Endocrinol.*, **3,** 372.

COFFEY, R.J., LEOF, E.B., SHIPLEY, G.D. & MOSES, H.L. (1987). Suramin inhibition of growth factor receptor binding and mitogenicity in AKR-2B cells. *J. Cell. Physiol.*, **132,** 143.

DATI, C., ANTONIOTTI, S., TAVERNA, D., PERROTEAU, I. & DE BORTOLI, M. (1990). Inhibition of c-*erb*B-2 oncogene expression by oestrogens in human breast cancer cells. *Oncogene* **5,** 1001.

DAVIDSON, N.E., GELMANN, E.P., LIPPMAN, M.E. & DICKSON, R.B. (1987). Epidermal growth factor receptor gene expression in estrogen receptor positive and negative human breast cancer cell lines. *Molec. Endocrinol.*, **1,** 216.

DERYNCK, R. (1988). Transforming growth factor α. *Cell*, **54,** 593.

DICKSON, R.B., BATES, S.E., MCMANAWAY, M.E. & LIPPMAN, M.E. (1986). Characterization of estrogen responsive transforming activity in human breast cancer cell lines. *Cancer Res.*, **46,** 1707.

FILMUS, J., POLLAK, M.N., CAILLEAU, R. & BUICK, R.N. (1985). MDA-468, a human breast cancer cell line with a high number of epidermal growth factor (EGF) receptors, has an amplified EGF receptor gene and is growth inhibited by EGF. *Biochem. Biophys. Res. Commun.*, **128,** 898.

FRAKER, P.J. & SPECK, J.C. (1978). Protein and cell membrane iodinations with a sparingly soluble chloramide, 1,3,4,6-tetrachloro-3a,6a-diphenylglycoluril. *Biochem. Biophys. Res. Commun.*, **80,** 849.

GINSBURG, E. & VONDERHAAR, B.K. (1985). Epidermal growth factor stimulates the growth of A431 tumors in athymic mice. *Cancer Lett.*, **28,** 143.

GRAHAM, M.L., KRETT, N.L., MILLER, L.A., LESLIE, K.K., GORDON, D.F., WOOD, W.M., WEI, L.L. & HORWITZ, K.B. (1990). T47D$_{co}$ cells, genetically unstable and containing estrogen receptor mutations, are a model for the progression of breast cancers to hormone resistance. *Cancer Res.*, **50,** 6208.

HAMBURGER, A.W. & PINNAMANENI, G.D. (1991). Increased epidermal growth factor gene expression by γ-interferon in a human breast carcinoma cell line. *Br. J. Cancer*, **64,** 64.

KEIGHTLY, D.D. & CRESSIE, N.A.C. (1980). The Woolf plot is more reliable than the Scatchard plot in analysing data from hormone receptor assays. *J. Steroid Biochem.*, **13,** 1317.

KNABBE, C., LIPPMAN, M.E., WAKEFIELD, L.M., FLANDERS, K.C., KASID, A., DERYNCK, R. & DICKSON, R.B. (1987). Evidence that transforming growth factor beta is a hormonally regulated negative growth factor in human breast cancer cells. *Cell*, **48,** 417.

LIPPMAN, M.E., DICKSON, R.B., GELMANN, E.P., ROSEN, N., KNABBE, C., BATES, S., BRONZERT, D., HUFF, K. & KASID, A. (1987). Growth regulation of human breast carcinoma occurs through regulated growth factor secretion. *J. Biochem.*, **35,** 1.

MARTIN, J.H.J., MCKIBBEN, B.M., LYNCH, M. & VAN DEN BERG, H.W. (1991). Modulation by oestrogen and progestins/antiprogestins of alpha interferon receptor expression in human breast cancer cells. *Eur. J. Cancer*, **27,** 143.

MURPHY, L.J., SUTHERLAND, R.L. & LAZARUS, L. (1985). Regulation of growth hormone and epidermal growth factor receptors by progestins in breast cancer cells. *Biochem. Biophys. Res. Commun.*, **131,** 767.

NELSON, J., MCGIVERN, M., WALKER, B., BAILIE, J.R. & MURPHY, R.F. (1989). Growth-inhibitory and growth-stimulatory effects of epidermal growth factor on human breast cancer cell line. MDA.MB.436 – Dependence on culture conditions. *Eur. Clin. Oncol.*, **25,** 1851.

NICHOLSON, S., HALCROW, P., SAINSBURY, J.R.C., ANGUS, B., CHAMBERS, P., FARNDON, J.R. & HARRIS, A.L. (1988*a*). Epidermal growth factor receptor (EGFr) status associated with failure of primary endocrine therapy in elderly postmenopausal patients with breast cancer. *Br. J. Cancer*, **58,** 810.

NICHOLSON, S., SAINSBURY, J.R.C., NEEDHAM, G.K., CHAMBERS, P., FARNDON, J.R. & HARRIS, A.L. (1988*b*). Quantitative assays of epidermal growth factor receptor in human breast cancer: cut-off points of clinical relevance. *Int. J. Cancer*, **42,** 36.

OSBORNE, C.K., HAMLITON, B., TITUS, G. & LIVINGSTON, R.B. (1980). Epidermal growth factor stimulation of human breast cancer cells in culture. *Cancer Res.*, **40,** 2361.

RIZZINO, A., RUFF, E. & KAZAKOFF, P. (1988). Isolation and characterisation of A-431 cells that retain high epidermal growth factor binding capacity and respond to epidermal growth factor by growth stimulation. *Cancer Res.*, **48,** 2377.

SAINSBURY, J.R.C., FARNDON, J.R., NEEDHAM, G.K., MALCOLM, A.J. & HARRIS, A.L. (1987). Epidermal growth factor receptor status as predictor of early recurrence of and death from breast cancer. *Lancet*, **i,** 1398.

SAVOURET, J.-F., FRIDLANSKI, F., ATGER, M., MISRAHI, M., BERGER, R. & MILGROM, E. (1991). Origin of the high constitutive level of progesterone receptor in T47-D breast cancer cells. *Mol. Cell. Endocrinol.*, **75,** 157.

VAN DEN BERG, H.W., LEAHEY, W.J., LYNCH, M., CLARKE, R. & NELSON, J. (1987). Recombinant human interferon alpha increases oestrogen receptor expression in human breast cancer cell (ZR-75-1) and sensitises them to the anti-proliferative effects of tamoxifen. *Br. J. Cancer*, **55,** 255.

VAN DEN BERG, H.W., LYNCH, M., MARTIN, J.H., NELSON, J., DICKSON, G.R. & CROCKARD, A.D. (1989). Characterisation of a tamoxifen resistant variant of the ZR-75-1 human breast cancer cell line, ZR-75-9a1 and stability of the resistant phenotype. *Br. J. Cancer*, **59,** 522.

VAN DEN BERG, H.W., MARTIN, J. & LYNCH, M. (1990). High progesterone receptor concentration in a variant of the ZR-75-1 human breast cancer cell line adapted to growth to oestrogen free conditions. *Br. J. Cancer*, **61,** 504.

ZOON, K.C., KARASAKI, Y., ZUNEDDEN, K., HU, R. & ARNHEITER, H. (1986). Modulation of epidermal growth factor receptors by human α interferon. *Proc. Natl Acad. Sci. USA*, **83,** 8226.