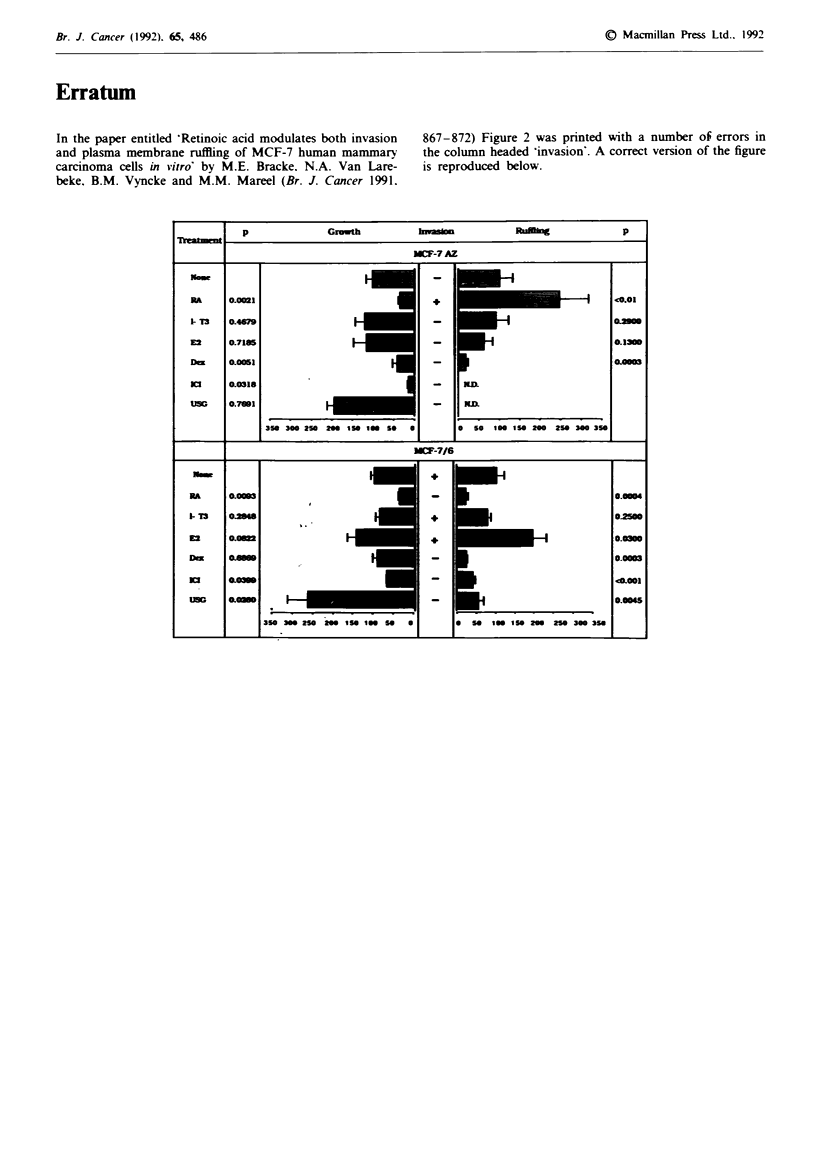# Erratum

**Published:** 1992-03

**Authors:** 


					
Br. J. Cancer (1992? 65, 486         ? Macmillan Press Ltd.. 1992~~~~~~~~~~~~~~~~~~~~~~~~~~~~~~~~~~~~~~~~~~~~~~~~~~~~~~~~~~~~~~~~~~~~~~~~~~~~~~~~~~~~~~~~~~~~~~~~~~~~~~~~~~~~~~~~~~~~~~~~~~~~~~~~~~~~~~~~~~~~~~~~~~~~~~~~~~~~~~~~

Erratum

In the paper entitled 'Retinoic acid modulates both invasion
and plasma membrane ruffling of MCF-7 human mammary
carcinoma cells in vitro' by M.E. Bracke. N.A. Van Lare-
beke B.M. Vyncke and M.M. Mareel (Br. J. Cancer 1991.

867-872) Figure 2 was printed with a number of errors in
the column headed 'invasion'. A correct version of the figure
is reproduced below.

p                 Growth                  i                                    p

NCF-7 AZ

RI      0-0021l                                                    1CO.l

I- 13  0t4579                                                                            0-2ge
E2     0Q7185                                      _ones
D       00051                                                                            eases
la:     0.0318                               *           [

US 0.7091                           _                    p

350 300 2S0 2"   ISO I"  S6   0         0   50  100 150 200 250 300 3SO

_   W-7/6

RA      0-0053                                                                             so
I-iS    0.2S4                                      +                                     s-2ss
E2       @.082                                                                           0.0000

Den  Me                                        _                                     0.0003

0S-OWe                                                                               s-rno

120     a.03_5                                                                           0.005

350S 2i.    Zn   5 so    @ s           0   S0  16  150 2S0 2S0 360 3S

C) Macmillan Press Ltd., 1991-

Br. J. Cancer (I 992). 65, 486